# Association of the *CPT1A* p.P479L Metabolic Gene Variant With Childhood Respiratory and Other Infectious Illness in Nunavut

**DOI:** 10.3389/fped.2021.678553

**Published:** 2021-07-06

**Authors:** Sorcha A. Collins, Sharon Edmunds, Gwen Healey Akearok, J. Robert Thompson, Anders C. Erickson, Elske Hildes-Ripstein, Amber Miners, Martin Somerville, David M. Goldfarb, Cheryl Rockman-Greenberg, Laura Arbour

**Affiliations:** ^1^Department of Medical Genetics, University of British Columbia, Vancouver, BC, Canada; ^2^Department of Research, Monitoring, and Evaluation, Nunavut Tunngavik Inc., Iqaluit, NU, Canada; ^3^Qaujigiartiit Health Research Centre, Iqaluit, NU, Canada; ^4^Cadham Provincial Laboratory, Winnipeg, MB, Canada; ^5^School of Population and Public Health, University of British Columbia, Vancouver, BC, Canada; ^6^Department of Pediatrics and Child Health, University of Manitoba, Winnipeg, MB, Canada; ^7^Department of Health, Government of Nunavut, Iqaluit, NU, Canada; ^8^Department of Laboratory Medicine and Pathobiology University of Toronto, Toronto, ON, Canada; ^9^Department of Pathology and Laboratory Medicine, University of British Columbia, Vancouver, BC, Canada

**Keywords:** Indigenous, Inuit, carnitine palmitoyltransferase 1A, infectious illness, p.P479L arctic variant, respiratory tract infection in children

## Abstract

**Objective:** Infectious illness, including lower respiratory tract infection (LRTI), is a leading cause of childhood morbidity and infant mortality in Inuit children in Nunavut Canada. The carnitine palmitoyltransferase 1A (CPT1A) p.P479L variant is common in arctic Indigenous populations of Alaska, Canada, and Greenland. CPT1A is a fatty acid oxidation enzyme expressed in the liver, immunocytes and other tissues, and is needed to use fats for energy during fasting. Previous association of the variant with early childhood infectious illness and infant death has been challenged because of sample size and limited adjustment for confounders. We evaluated whether the p.P479L variant is associated with infectious illness in Inuit children of Nunavut, Canada.

**Methods:** We conducted a retrospective clinical chart review of 2,225 Inuit children (0–5 years) for infectious illness (including otitis media, gastroenteritis, and hospital admission for LRTI), prenatal, perinatal, and socioeconomic indicators, subsequently linking to *CPT1A* genotype. Multivariable logistic regression adjusted for birth characteristics, breastfeeding, maternal smoking, food insecurity, and socioeconomic indicators.

**Results:** Overall, 27% of children were hospitalized for LRTI, 86% had otitis media and 50% had gastroenteritis. The p.P479L allele frequency was 0.82. In multivariable analysis, p.P479L homozygosity was associated with LRTI admission (aOR:2.88 95%CI:1.46–5.64), otitis media (aOR:1.83, 95%CI:1.05–3.21), and gastroenteritis (aOR:1.74, 95%CI:1.09–2.77), compared to non-carriers.

**Conclusion:** Children homozygous for the p.P479L variant were more likely to experience infectious illness than non-carriers, including hospitalization for respiratory tract infections. Given the role of CPT1A in immunocytes, our findings indicate that more study is needed to determine if there is a role of the variant in immune response. Continued Inuit involvement is essential when considering next steps.

## Introduction

Infants and children in Nunavut have high rates of infectious illness, in particular, infant hospitalizations for lower respiratory tract infections (LRTI; 234-306/1000) (1, 2) and otitis media (85% of children <3 years) (3). Nunavut also has an infant mortality rate four times the national average (21.5 vs. 4.5/1,000 livebirths) (4), with sudden unexpected death and respiratory infection comprising the majority of post-neonatal deaths (5). Nunavut is Canada's northernmost territory and largest jurisdictional landmass, which is divided into three regions, Qikiqtani, Kivalliq, and Kitikmeot. The majority of Nunavut residents (referred to as Nunavummiut) identify as Inuit (80% of 40,000 inhabitants) (6, 7), with unique socio-cultural strengths and perspectives. Although social determinants of health and institutional structures that reinforce socioeconomic status influence health outcomes (3, 8, 9), underlying genetic factors may also play a role. Studies have reported that the p.P479L variant of carnitine palmitoyltransferase 1A (CPT1A) may contribute to the high rates of infectious illness and infant death in Northern Indigenous populations (5, 10–12).

Nunavut has a high prevalence of the p.P479L (c.1436C>T, rs80356779) variant of CPT1A, which is prevalent in Indigenous populations of northern Canada, Alaska and Greenland (13–18). The variant has been associated with a number of adverse infant and child health outcomes, including hypoglycemia (10, 19, 20), seizures (21) and sudden unexpected infant death and infant death due to infection (5, 13, 22). Preliminary studies have suggested the variant may be also be associated with infectious illness in early childhood (11, 12). CPT1A is the rate limiting enzyme of long chain fatty acid oxidation in a variety of tissues, including the liver, pancreas and immunocytes (23). Classic CPT1A deficiency (OMIM:255120) is a rare autosomal recessive disease presenting during infancy as hypoketotic hypoglycemia and metabolic decompensation triggered by prolonged fasting and/or vomiting, often precipitated by active infection (24). Unlike variants that cause classic CPT1A deficiency that abolish CPT1A activity, the CPT1A p.P479L variant, also known as the “arctic variant,” is considered a mild variant with reported CPT1A activity of 2–22% of normal (10, 25).

Given the p.P479L variant's high frequency, there has been uncertainty regarding its clinical significance, which has created calls for larger and more comprehensive studies that include other risk factors important in these outcomes (26). We assessed early child health outcomes of 2,225 Inuit children residing in Nunavut to determine whether the CPT1A p.P479L variant is associated with infectious illness in the broader context of other relevant perinatal, postnatal and socioeconomic variables.

## Methods

### Ethics Statement

The study was developed and conducted in partnership with Nunavut Tunngavik Inc. (NTI), the Qaujigiartiit Health Research Centre (QHRC) and the Government of Nunavut Department of Health. NTI is responsible for ensuring the implementation of and adherence to the Nunavut Land Claims Agreement and advocates for policies and programs that enhance Inuit well-being, including healthy children. The QHRC is a community-led research institute, fostering local leadership and engagement in research activities involving the health and well-being of Nunavummiut.

Study ethics approval was granted by the University of British Columbia, University of Victoria and University of Manitoba Research Ethics Boards, and a research license was granted by the Nunavut Research Institute.

### Data Sources

We reviewed clinical charts of Inuit children born from 01-Jan-2010 to 31-Dec-2013 at community health centers, Iqaluit Public Health and the Qikiqtani General Hospital in Iqaluit. All communities with more than 20 births/year (18/25 communities) were visited for chart review in all three Nunavut regions (Qikiqtaaluk, Kivalliq, and Kitikmeot). Information collected included birth data (gestational age, birth weight, place and type of birth, complications, newborn screening results), perinatal and postnatal exposures from prenatal, labor/delivery, newborn and well-baby records and medical information up to 5 years of age (0–5 years). Medical data abstracted included reason for visit/admission, tests, treatments, outcomes, and medical diagnoses.

Inuit ethnicity was determined using mother's and/or infant's ancestry indicated on the chart. Food insecurity data were collected as recorded on the well-baby records for 2, 6, 12, 24, and 48-month visits using the primary caregiver answer to the question “since your baby was born/your last visit, were there times when the food for you and your family just did not last and there was no money to buy enough food?” Answers of “Often” or “Sometimes” were coded as Yes, “No/Never” as No, and “Don't know/refused” as missing. Breastfeeding data was collected from newborn records and well-baby records for 1 week−1 month, 2, 6, 12, and 24 months visits.

Community SES was defined using the 2011 Statistics Canada Community Well-Being (CWB) index that provides a measure of socioeconomic well-being for individual communities across Canada (27). The CWB is comprised of four components (education, labor force activity, income, and housing) combined into an index between 0 (lowest) and 100 (highest).

Outcome variables were admission for LRTI (>24 h to regional/tertiary hospital), admission with respiratory syncytial virus (RSV; >24 h to regional/tertiary hospital for laboratory confirmed RSV infection), otitis media, gastroenteritis (vomiting and/or diarrhea not otherwise explained) and dental interventions (restorations, extractions, treatment of infection, surgery). Repeat visits/admissions within 14 days were not counted.

#### CPT1A p.P479L Genotyping

Retrospective *CPT1A* p.P479L (rs80356779) genotyping was conducted for all infants born to mothers residing in Nunavut between Jan 1, 2010 to Dec 31, 2013 by re-testing stored newborn dried blood spot cards. Testing was conducted by the newborn screening programme at Cadham Provincial Laboratory in Winnipeg Manitoba for all Kivalliq region births, as previously described (10), and by Newborn Screening Ontario at the Children's Hospital of Eastern Ontario for in Kitikmeot and Qikiqtaaluk births, as previously described (14). Genotype results were linked to the clinical information after the completion of the chart review. As a retrospective anonymized study, no CPT1A p.P479L results were returned to health care providers or families. A small portion of children in the study cohort had been tested for the p.P479L variant as part of a pilot newborn screening program. In the case of a positive result (p.P479L homozygous), the result was reported by letter to community health care providers advising that the clinical significance of the variant was uncertain but to avoid prolonged fasting. This information was collected from the clinical chart during the chart review.

### Statistical Analysis

We used descriptive statistics to summarize differences in covariates and outcomes by CPT1A p.P479L variant status. We conducted univariable tests of statistical significance using logistic and linear regression to explore relationships between variables and outcomes and pairwise correlation tests to show the inter-relationships between the variables. We used complete case multivariable logistic regression to examine association of CPT1A p.P479L variant with outcomes using two models: Model 1 adjusted for CWB and residence in Iqaluit. Model 2 adjusted for CWB, residence in Iqaluit, sex, preterm birth (PTB; <37 weeks gestation), presence of major congenital anomalies (included in LRTI and RSV admission models only), postnatal maternal smoking, breastfeeding ≥6 months and food insecurity. Odds ratios with 95% confidence intervals were considered statistically significant for two-tailed *p*-values < 0.05.

We conducted sensitivity analysis using multiple imputation by chained equations to create missing values for preterm birth (*n* = 34), postnatal maternal smoking (*n* = 408), breastfeeding ≥6 months (*n* = 145), and food insecurity (*n* = 455). All variables and outcomes were included in the imputation and 20 imputed data sets were created. Comparison of imputed data to complete case analysis was then performed. To investigate whether prior knowledge of p.P479L homozygosity impacted study results, we conducted a sensitivity analysis by running models 1 and 2 with (1) excluding cases with prior documented p.P479L homozygosity and (2) including a covariate for prior documented p.P479L homozygosity. Results were compared to complete case analysis.

Hardy-Weinberg equilibrium (HWE) was analyzed using the χ^2^ test with *p* < 0.05 significance level for each region and the territorial capital Iqaluit, which has the largest population in Nunavut. Data were analyzed using Stata Statistical Software: Release 16SE (StataCorp LLC).

## Results

Charts for 2,523 Inuit children were reviewed. Charts with only birth data were excluded from analysis (*n* = 60). *CPT1A* genotype linkage was successful for 2,225/2,463 records (90.3%). Of those, 68.7% were p.P479L homozygous, 25.6% p.P479L heterozygous and 5.7% non-carrier wildtype (Table 1). The p.P479L allele was in HWE in Kitikmeot and the territorial capital (Iqaluit) but not Kivalliq, Qikiqtaaluk or for the population of Nunavut as a whole. Of the 2,225 children with p.P479L status information, 140 had p.P479L homozygous positive test results documented in the chart.

**Table 1:** Regional distribution of CPT1A p.P479L variant in Inuit children born in Nunavut (2010–2013, *n* = 2,225).

	**Total**	**Non-carrier wildtype**		**p.P479L Heterozygous**		**p.P479L Homozygous**		**p.P479L allele 2010–13 Inuit births**		**p.P479L allele 2006 births^a^**		
	***n***	***n***	**freq**	***n***	**Freq**	***n***	**freq**	**freq**	**(95%CI)**	***n***	**freq**	**(95%CI)**
Nunavut	2,225	126	0.057	570	0.256	1,529	0.687	0.815^b^	(0.805–0.828)	695	0.770^b^	(0.747–0.792)
Kitikmeot	482	11	0.023	98	0.203	373	0.774	0.876	(0.853–0.896)	150	0.850	(0.804–0.888)
Kivalliq	754	51	0.068	214	0.284	489	0.649	0.791^b^	(0.769–0.811)	243	0.827	(0.791–0.860)
Qikiqtaaluk	989	64	0.065	257	0.260	668	0.675	0.805^b^	(0.788–0.823)	302	0.684^b^	(0.645–0.721)
Iqaluit	250	40	0.160	110	0.440	100	0.400	0.620	(0.576–0.663)		n/a	

a *Data from all live births in 2006 to women residing in Nunavut (14)*.

b *Allele frequency not in Hardy-Weinberg equilibrium, 95%CI, 95% Confidence interval*.

There were no significant differences in birth related characteristics between *CPT1A* genotype groups (Table 2). Pairwise correlation analyses showed p.P479L homozygosity was positively correlated with all health outcomes assessed, postnatal maternal smoking and food insecurity and was negatively correlated with CWB and residence in Iqaluit (Supplementary Table 1).

**Table 2:** Infant and maternal characteristics by CPT1A p.P479L variant for Inuit children born in Nunavut (2010–2013, *n* = 2,225).

	**Non-carrier wildtype (*n* = 126)**	**p.P479L heterozygous (*n* = 570)**	**p.P479L homozygous (*n* = 1,529)**	**Total cohort (*n* = 2,225)**	**Missing**
	***n* /total (%)**	***n* /total (%)**	***n* /total (%)**	***n* /total (%)**	***n* (%)**
Male	58/126 (46.0)	289/570 (50.7)	773/1,529 (50.6)	1,120/2,225 (50.3)	0
Preterm (<37 weeks)	13/124 (10.5)	67/565 (12.0)	186/1,502 (12.4)	266/2,191 (12.1)	34 (1.5)
mean GA	38.7 weeks	38.5 weeks	38.2 weeks	38.1 weeks	
Term LBW (<2,500 g)	0/123 (0)	6/560 (1.1)	33/1,513 (2.2)	39/2,196 (1.8)	29 (1.3)
mean birth weight	3,526 g	3,456 g	3,344 g	3,341 g	
Mat. age <20 years	26/117 (22.2)	98/529 (18.5)	331/1,421 (23.3)	455/2,067 (22.0)	158 (7.1)
Mean mat. age	24 years	24 years	24 years	24 years	
Breastfeeding ≥6 months	43/123 (35.0)	200/531 (37.7)	490/1,426 (34.4)	733/2,080 (35.2)	145 (6.5)
Postnatal mat smk.	61/94 (64.9)	384/460 (83.4)	1,119/1,263 (88.6)	1,564/1,817 (86.1)	408 (18.3)
Food insecurity <5 years	21/93 (22.6)	149/450 (33.1)	577/1,227 (47.0)	747/1,770 (42.2)	455 (20.4)

*CPT1A, Carnitine palmitoyltransferase 1A; Total, number of charts with data for category; Term LBW, Term low birth weight (37 weeks gestation, <2,500 g), Mat; Age, maternal age; Postnatal mat smk., Postnatal maternal smoking; GA, Gestational age*.

Overall, 607 (27.3%) children were admitted to regional or tertiary hospital for LRTI, including 182 (8.2%) admitted with RSV. The majority of children had one or more episodes of otitis media (86%) and half of all children (50%) had at least one episode of gastroenteritis (Table 3).

**Table 3:** Infectious illness by CPT1A p.P479L variant in Inuit children born in Nunavut (2010–2013, *n* = 2,225).

	**Cohort (*n* = 2,225)**	**Non-carrier (*n* = 126)**	**p.P479L heterozygous (*****n*** **=** **570)**		**p.P479L homozygous (*****n*** **=** **1,529)**	
**Outcome variable**	***n* (%)**	***n* (%)**	***n* (%)**	**cOR (95%CI)**	***n* (%)**	**cOR (95%CI)**
LRTI admitted, 0–5 years	607 (27.3)	15 (11.9)	105 (18.4)	1.7 (0.93–3.0)	487 (31.9)	3.5 (2.0–6.0)
LRTI admitted, infants (<1 year)	449 (20.2)	9 (7.1)	71 (12.5)	1.9 (0.89–3.8)	369 (24.1)	4.2 (2.1–8.3)
RSV admitted, 0–5 years	182 (8.2)	3 (2.4)	39 (6.8)	3.0 (0.92–9.9)	140 (9.2)	4.1 (1.3–13.2)
RSV admitted, infants (<1 year)	149 (6.7)	3 (2.4)	31 (5.4)	2.4 (0.71–7.9)	115 (7.5)	3.3 (1.0–10.7)
Otitis media, 0–5 years	1,919 (86.3)	90 (71.4)	474 (83.2)	2.0 (1.3–3.1)	1,355 (88.6)	3.1 (2.1–4.7)
Otitis media 3+ episodes, 0–5 years	1,290 (57.2)	48 (37.8)	274 (46.8)	1.4 (0.98–2.1)	954 (62.4)	2.8 (1.9–4.1)
Otitis media, infants (<1 year)	1,413 (63.5)	54 (42.9)	320 (56.1)	1.7 (1.2–2.5)	1,039 (67.9)	2.8 (2.0–4.1)
Gastroenteritis, 0–5 years	1,109 (49.8)	47 (37.3)	249 (43.7)	1.3 (0.87–1.9)	813 (53.1)	1.9 (1.3–2.8)
Gastroenteritis, infants (<1 year)	637 (28.6)	19 (15.1)	137 (24.0)	1.8 (1.1–3.0)	481 (31.5)	2.6 (1.6–4.3)
Dental interventions, 0–5 years	794 (35.7)	22 (17.5)	188 (33.0)	2.3 (1.4–3.8)	584 (38.2)	2.9 (1.8–4.7)
**Mean num. admits/illnesses**	**Mean (95% CI)**	**Mean (95% CI)**	**Mean (95% CI)**	**Coef. (*****p*****)**	**Mean (95% CI)**	**Coef. (*****p*****)**
LRTI admits, 0–5 years	0.42 (0.38–0.46)	0.15 (0.07–0.23)	0.24 (0.20–0.29)	0.09 (0.278)	0.51 (0.46–0.56)	0.36 (<0.001)
mean age 1st admit (months)	9.0 (8.1–9.9)	15.4 (5.5–25.2)	12.0 (9.3–14.6)	−3.4 (0.280)	8.6 (7.7–9.6)	−6.7 (0.025)
Otitis media, 0–5 years	4.1 (3.9–4.3)	2.4 (1.9–2.8)	3.0 (2.8–3.3)	0.62 (0.11)	4.6 (4.4–4.9)	2.3 (<0.001)
mean age at 1st episode (months)	11.6 (11.2–12.1)	13.6 (11.2–16.2)	13.6 (12.5–14.7)	−0.02 (0.988)	10.8 (10.3–11.3)	−2.8 (0.015)
Gastroenteritis, 0–5 years	0.93 (0.88–0.99)	0.61 (0.43–0.79)	0.73 (0.64–0.81)	0.12 (0.35)	1.0 (0.97–1.1)	0.43 (<0.001)
mean age at 1st episode (months)	14.1 (13.4–14.8)	17.4 (13.6–21.2)	14.5 (12.9–16.0)	−2.9 (0.123)	13.8 (13.0–14.6)	−3.6 (0.044)

*CPT1A, carnitine palmitoyltransferase 1A; cOR, crude odds ratio; CI, confidence interval; Coef, regression coefficient; LRTI, lower respiratory tract infection; RSV, Respiratory syncytial virus; Dental, major dental interventions (extractions, restorations, surgeries)*.

Results of univariable and multivariable logistic regression analysis are presented in Table 4. In univariable logistic regression analysis, p.P479L homozygosity was associated with all outcomes and p.P479L heterozygosity was associated with otitis media, dental interventions and gastroenteritis during infancy (Table 4). In multivariable regression analysis adjusting for CWB and residence in Iqaluit (Model 1), p.P479L homozygosity was associated with all outcomes except RSV admission in infancy and p.P479L heterozygosity was associated with otitis media and dental interventions in early childhood but not gastroenteritis during infancy.

**Table 4:** Multivariable logistic regression results for association of CPT1A p.P479L variant with infectious illness during infancy and early childhood in Inuit children residing in Nunavut (2010–2013).

	**Early childhood (0–5 years)**				**Infants (<1 year)**			
	**p.P479L homozygous**		**p.P479L heterozygous**		**p.P479L homozygous**		**p.P479L heterozygous**	
	**OR (95%CI)**	***p***	**OR (95%CI)**	***P***	**OR (95%CI)**	***P***	**OR (95%CI)**	***p***
**LRTI admission**								
Univariable	3.47 (2.00–6.01)	**<0.001**	1.66 (0.93–2.95)	0.082	4.15 (2.08–8.25)	**<0.001**	1.82 (0.89–3.76)	0.103
Model 1^a^	3.19 (1.82–5.60)	**<0.001**	1.62 (0.90–2.90)	0.101	3.28 (1.63–6.58)	**0.001**	1.64 (0.79–3.39)	0.182
Model 2 (cc)^b^	2.88 (1.46–5.64)	**0.002**	1.63 (0.81–3.29)	0.169	2.79 (1.29–6.03)	**0.009**	1.54 (0.69–3.44)	0.291
Model 2 (imputed)^b^	3.11 (1.75–5.52)	**<0.001**	1.64 (0.91–2.98)	0.102	3.26 (1.60–6.64)	**0.001**	1.69 (0.81–3.54)	0.161
**RSV admission**								
Univariable	4.13 (1.30–13.15)	**0.016**	3.02 (0.92–9.92)	0.069	3.33 (1.04–10.64)	**0.042**	2.36 (0.71–7.85)	0.161
Model 1^a^	4.17 (1.29–13.47)	**0.017**	3.07 (0.93–10.13)	0.066	2.89 (0.89–9.36)	0.077	2.23 (0.67–7.43)	0.193
Model 2 (cc)^b^	3.04 (0.92–10.07)	0.068	2.61 (0.77–8.82)	0.122	2.02 (0.61–6.71)	0.249	1.79 (0.52–6.11)	0.355
Model 2 (imputed)^b^	4.12 (1.27–13.41)	**0.019**	3.11 (0.94–10.32)	0.064	2.81 (0.86–9.18)	0.087	2.23 (0.66–7.47)	0.194
**Otitis media**								
Univariable	3.12 (2.05–4.73)	**<0.001**	1.97 (1.26–3.07)	**0.003**	2.83 (1.96–4.09)	**<0.001**	1.70 (1.15–2.51)	**0.008**
Model 1^a^	1.95 (1.25–3.06)	**0.004**	1.64 (1.03–2.61)	**0.036**	1.83 (1.23–2.70)	**0.003**	1.41 (0.94–2.12)	0.096
Model 2 (cc)^c^	1.83 (1.05–3.21)	**0.034**	1.67 (0.94–2.99)	0.081	1.87 (1.18–2.96)	**0.008**	1.53 (0.95–2.47)	0.084
Model 2 (imputed)^c^	1.96 (1.24–3.10)	**0.004**	1.64 (1.02–2.62)	**0.040**	1.90 (1.28–2.82)	**0.002**	1.44 (0.95–2.17)	0.082
**Gastroenteritis**								
Univariable	1.91 (1.31–2.78)	**0.001**	1.30 (0.87–1.93)	0.197	2.58 (1.57–4.26)	**<0.001**	1.79 (1.06–3.02)	**0.030**
Model 1^a^	1.62 (1.10–2.38)	**0.015**	1.21 (0.81–1.81)	0.344	2.00 (1.20–3.34)	**0.008**	1.61 (0.95–2.73)	0.078
Model 2 (cc)^c^	1.74 (1.09–2.77)	**0.020**	1.32 (0.81–2.13)	0.264	2.32 (1.23–4.39)	**0.010**	2.01 (1.04–3.87)	**0.037**
Model 2 (imputed)^c^	1.65 (1.11–2.44)	**0.013**	1.24 (0.83–1.86)	0.302	2.00 (1.19–3.36)	**0.009**	1.62 (0.95–2.77)	0.075
**Dental interventions**								
Univariable	3.14 (1.98–5.00)	**<0.001**	2.37 (1.46–3.84)	**<0.001**				
Model 1^a^	2.23 (1.38–3.58)	**0.001**	2.06 (1.26–3.36)	**0.004**				
Model 2 (cc)^c^	2.11 (1.22–3.66)	**0.008**	1.88 (1.07–3.32)	**0.029**				
Model 2 (imputed)^c^	2.27 (1.41–3.67)	**0.001**	2.09 (1.27–3.41)	**0.003**				

a *Adjusted for community socioeconomic status (CWB) and residence in Iqaluit*.

b *Adjusted for community socioeconomic status (CWB) and residence in Iqaluit, sex, preterm birth (<37 weeks gestation), major congenital anomalies, postnatal maternal smoking, breastfeeding ≥6 months, and food insecurity*.

c *Adjusted for community socioeconomic status (CWB) and residence in Iqaluit, sex, preterm birth, postnatal maternal smoking, breastfeeding ≥6 months and food insecurity*.

*CPT1A, carnitine palmitoyltransferase 1A; OR, odds ratio; CI, confidence interval; LRTI, lower respiratory tract infection; RSV, respiratory syncytial virus; Dental, major dental interventions (extractions, restorations, surgeries); cc, complete case analysis, n = 1,697*.

*Bold values indicate two-tailed p < 0.05*.

**Figure 1:**
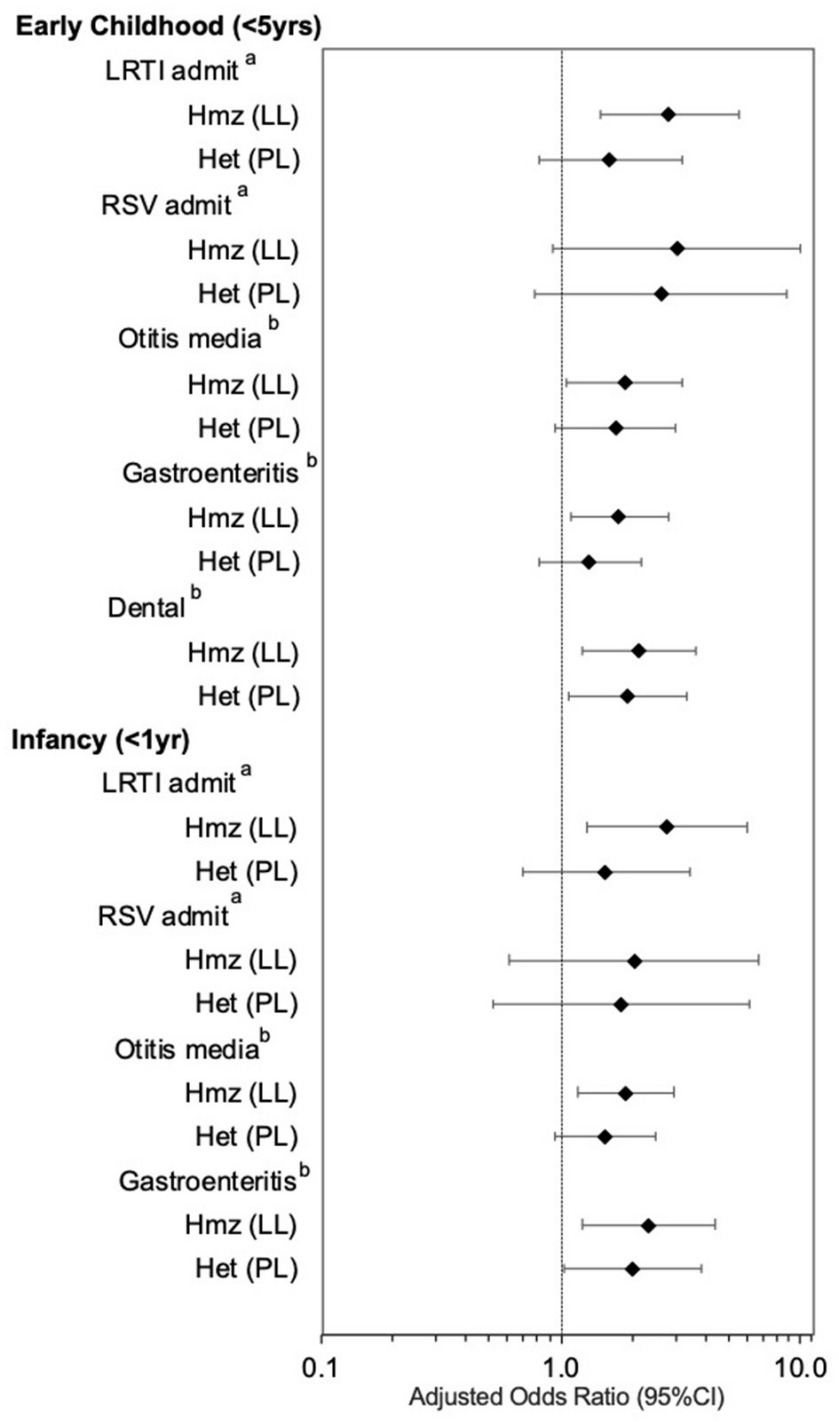
CPT1A p.P479L variant and infectious illness, by age group, adjusting for CWB and Iqaluit residence, sex, preterm birth, major congenital anomalies (LRTI and RSV), maternal smoking, breastfeeding 6 months+, and food insecurity. Hmz, homozygous; Het, heterozygous; LRTI, lower respiratory tract infection; RSV, Respiratory syncytial virus; OM, Otitis media. ^a^Adjusted for community socioeconomic status (CWB) and residence in Iqaluit, sex, preterm birth (<37 weeks gestation), major congenital anomalies, postnatal maternal smoking, breastfeeding 6 months or longer, and food insecurity. ^b^Adjusted for community socioeconomic status (CWB) and residence in Iqaluit, sex, preterm birth, postnatal maternal smoking, breastfeeding 6 months or longer, and food insecurity.

Figure 1 shows the effect estimates for p.P479L homozygosity and heterozygosity after further adjustment for postnatal and socioeconomic variables (Model 2). p.P479L homozygosity was associated with LRTI admission (aOR:2.88 95%CI:1.46-5.64), otitis media (aOR:1.83, 95%CI:1.05–3.21), gastroenteritis (aOR:1.74, 95%CI:1.09–2.77), and dental intervention (aOR:2.11, 95%CI:1.22–3.66) in early childhood and p.P479L heterozygosity was associated with dental interventions (aOR:1.88, 95%CI:1.07–3.32; Figure 1). In infancy (<1 year of age), p.P479L homozygosity was associated with LRTI admission (aOR:2.79, 95%CI:1.29–6.03), otitis media (aOR:1.87, 95%CI:1.18–2.96), and gastroenteritis (aOR:2.32, 95%CI:1.23–4.39) and p.P479L heterozygosity was associated with gastroenteritis (aOR:2.01, 95%CI:1.04–3.87).

### Sensitivity Analysis

To understand the impact of missing data in the study, we conducted multiple imputation and compared model results to complete case analysis. All statistically significant associations with p.P479L homozygosity were retained, and the effect estimates for p.P479L homozygosity with RSV admission and p.P479L heterozygosity with otitis media in early childhood no longer overlapped one (Table 4). However, the association of p.P479L heterozygosity with gastroenteritis in infancy was no longer statistically significant. We conducted a sensitivity analysis to investigate whether prior knowledge of p.P479L homozygosity impacted study results. All statistically significant associations between p.P479L homozygosity and outcomes were retained when records with documented p.P479L homozygosity were excluded from models (*n* = 140) or when models included adjustment for this information. Although there was slight increase or decrease of point estimates for the association of p.P479L homozygosity with some outcomes, there was no overall impact on the results of the study (see Supplementary Table 2).

## Discussion

The health status of Inuit children in Canada has garnered attention and concern for several decades (1, 28) and Inuit children living in Nunavut have some of the highest reported rates of infectious illness in Canada (1–3). Preliminary studies have implicated the CPT1A p.P479L variant as a possible risk factor for infectious illness in early childhood (11, 12); however, there has been some debate regarding the clinical significance of the variant and calls for larger, population-based comprehensive studies that adjust for known and suspected risk factors for adverse health outcomes (26).

In our comprehensive retrospective study of 2,225 Inuit children, the largest to date, we show that children homozygous for the p.P479L variant had significantly higher rates of infectious illness than non-carriers, which was independent of sex, preterm birth, residence in Iqaluit, breastfeeding for 6 months or longer, postnatal maternal smoking, food insecurity and CWB index (which includes measures of community-level housing, education, and income). Children homozygous for the variant were almost three times as likely to be admitted to hospital for LRTI. We noted that although p.P479L homozygosity was associated with early childhood RSV admission in Model 1 (CWB index and Iqaluit residence) confidence intervals in the full model overlapped one. Since RSV test results were not available for all LRTI admissions, this study may underrepresent the true prevalence of RSV admission, limiting the interpretation of this result.

Children homozygous for the p.P479L variant were also approximately twice as likely to have otitis media and gastroenteritis and require dental interventions than non-carriers in early childhood. Otitis media is associated with impaired hearing at 5 years of age and can have dramatic impacts on speech development and educational attainment (29–31). There are a number of risk factors associated with otitis media; including maternal smoking and preterm birth; however, the association of p.P479L variant with otitis media remained statistically significant after adjustment for many of those factors.

Our results corroborate and are consistent with studies in British Columbia and Alaska reporting association of the p.P479L variant with hospitalization and infectious illness. In 2013, Gessner et al. (11) reported that p.P479L homozygosity was associated with increased risk of otitis media (aOR:3.0, 95%CI:1.8–5.1) and LRTI admission (aOR:2.5, 95%CI:1.6–4.0) and admission for any reason in 427 Alaska Native children (0–2.5 years), adjusting for maternal education, age, prenatal smoking and alcohol use, prenatal care and birth weight. In 2019, Sinclair et al. (12) found that p.P479L homozygosity was associated with admissions for LRTI (OR:6.0, 95%CI:1.6–22.4), otitis media (OR:13.5, 95%CI:1.5–109.4) and dental caries (OR:3.4, 95%CI:1.5–7.8) in 150 children (<7 years) in a British Columbia First Nations community. In our current analysis, we demonstrate that adjustment for SES indicators, including food security and community level measures of education and housing, reduced but did not abolish the association with LRTI admission, otitis media and dental interventions.

Children homozygous for the CPT1A p.P479L variant may experience a more severe illness due to impaired ketogenesis or impaired response of the immune system during infection. As an enzyme critical for long chain fatty acid oxidation, CPT1A is important in a number of tissues, including liver energy homeostasis during fasting, pancreatic glucagon secretion and T cell development and survival (32–34). CD8+ memory T cells (32), especially resident memory T cells (33), and CD4+ Th17 and Treg (34) cells have high demands for fatty acid oxidation and CPT1A activity. Currently, there is no evidence regarding whether the p.P479L variant impairs immune response and/or memory T cell response to repeat infection in humans; however, a recent study of a multiple sclerosis (MS) mouse model (autoimmune encephalomyelitis) found that the CPT1A p.P479L variant may affect the function of cells important in immunity (35). The authors reported that knock-in mice expressing the *Cpt1a* p.P479L gene variant were resistant to the induction of autoimmune encephalomyelitis, suggesting that the variant conferred protection through blocking the activation of lipid metabolism and/or through impairing the activation of immune system (35). In the context of our study, these results suggest that there should be further studies on the potential effect of altered CPT1A function on immunity to determine if there is evidence of T-cell impact and subsequent impact on vaccine effectiveness for those with the CPT1A p.P479L variant.

The high prevalence of the p.P479L variant in Inuit populations, where the variant flourished to become the major allele (14, 36), suggests an historical advantage compared to non-carriers (18) likely due to synergy with traditional diet practices (10, 15). This speculation is supported by reports of strong signals of positive selection at the site of the nucleotide change (rs80356779) (16–18) and the association of the variant with protection from adverse lipid profiles in adults, including higher HDL-cholesterol and ApoA1 and lower adiposity (15, 36). There is also evidence of an interaction between traditional food intake and the p.P479L variant, supporting the possibility that this interaction may have influenced selection (37). The p.P479L variant is moderately insensitive to malonyl-CoA with fed state residual activity four times control (0.094 vs. 0.023 nmol/min/mg) (25), suggesting a degree of fatty acid metabolism occurs even when carbohydrate is present. Within the harsh arctic environment, this may have conferred advantage for those utilizing a traditional “hunter's diet” rich in omega-3 marine-based fats with little carbohydrate (10, 15).

In our study, p.P479L heterozygosity was associated with dental interventions, gastroenteritis in infancy and with otitis media in Model 1 (adjusting for CWB index and Iqaluit residence), indicating a possible heterozygous effect and could indicate an additive effect for the variant (Tables 3, 4). These results are thought-provoking since classic CPT1A deficiency is considered an autosomal recessive disorder. Other studies have reported intermediate results for p.P479L heterozygotes. In their study of adult Greenlanders, Skotte et al. (16) reported an association between the p.P479L variant and height with an average reduction in height of 2.1 cm per copy of the variant (*p* = 1.04 × 10^−9^), which suggests an additive effect. Furthermore, although the associations were not statistically significant, Sinclair et al. (12) found p.P479L heterozygotes had an intermediate effect (between wildtype and homozygotes) for LRTI admission and dental caries. Rajakumar et al. (36) also found an intermediary heterozygote effect on HDL-cholesterol and associated apoA-I levels. Taken together, these results support a possible additive effect in these outcomes.

Tobacco smoke exposure, household overcrowding and food insecurity are associated with childhood infectious illness (38, 39). In the current cohort, 86% of women reported smoking postnatally and 17.5% of children lived in homes with three or more people/bedroom. In pairwise correlation tests, p.P479L homozygosity was significantly correlated with maternal postnatal smoking, food insecurity and CWB, suggesting that some risk associated with the p.P479L variant may be due to these underlying factors; however, p.P479L homozygosity remained significantly associated with LRTI admission, otitis media, gastroenteritis and dental interventions after adjustment for these variables. Breastfeeding is associated with reduced risk for adverse early child health outcomes, including infant mortality and infectious illness (2, 40). Health Canada recommends breastfeeding for at least 6 months since longer duration of breastfeeding is protective against infectious illness in infancy (41); however, adjustment for breastfeeding 6 months or longer did not impact the association of p.P479L homozygosity with infectious illness outcomes.

The results of this study need to be interpreted in the context of the population where the study was conducted, which, as demonstrated in this study, has significant challenges with housing, food security and access to secondary and tertiary medical care due to the remote nature of Nunavut communities. Of consideration, genetic research in Indigenous communities may inadvertently cause harm by creating stigma for those with a described “risk” allele (9). However, there is also harm in avoiding research that may provide insights into health disparity. Health related research, and in particular, genetic research, is best carried out in partnership with Indigenous people to ensure that the balance of harms and benefits are fully considered throughout the process (42). It is important to note that this research was carried out with the support and on-going partnership of the Inuit organization that is responsible for the land and the people of Nunavut (NTI), and the grass roots Inuit research organization that leads relevant health research within the territory (QHRC). Next steps to address the impact of the p.P479L variant need to be developed in partnership with Inuit leadership and engagement with communities.

## Limitations

This was a retrospective chart review study. In the study cohort, 9% of records did not have *CPT1A* genotype information and were excluded from analysis. The CPT1A p.P479L variant was the only genetic variant studied; we cannot rule out that other genetic variants may have contributed to the results. The p.P479L variant departed from HWE in two Nunavut regions, which may be due to positive selection for the variant (16–18) since selection can cause deviations of HWE, or may be due to other unknown contributors or underlying differences in population structure. To mitigate the latter risk, the analysis was limited to Inuit children (as recorded on chart after self-identification); however, we were not able to assess non-Inuit admixture within the self-identified Inuit population.

## Conclusion

Children homozygous for the p.P479L variant were more likely to be admitted for lower respiratory tract infections and were more likely to have otitis media, gastroenteritis and dental interventions, supporting a role of the variant in infant and child health and well-being. Our results have international implications given the known prevalence of the p.P479L variant in circumpolar populations and should be further addressed with the goal of reducing infant and child morbidity and mortality. Further studies to determine if the CPT1A p.P479L variant impacts immune response to infection are needed, information that will be important for the development of culturally relevant public health strategies in reducing childhood morbidity and mortality.

## Data Availability Statement

The data analyzed in this study is subject to the following licenses/restrictions: UBC-UVic-Government of Nunavut Research Disclosure Agreement M17-00229. Requests to access these datasets should be directed to Dr. Laura Arbour, larbour@uvic.ca.

## Author Contributions

SC: participated in all aspects of the study including study conception and design, acquisition, analysis and interpretation of data, drafting of the initial manuscript, and revision of the manuscript. CR-G: participated in the study conception and design, acquisition, and interpretation of data along with revision of the manuscript. JRT: participated in design and acquisition of study data along with revision of the manuscript. SE, AE, EH-R, DG, AM, and MS: participated in the study design and interpretation of the data along with revision of the manuscript. LA: participated in the study conception and design, analysis, interpretation of data, and revision of the manuscript. All authors approved the final manuscript as submitted and agree to be accountable for all aspects of the work.

## Conflict of Interest

The authors declare that the research was conducted in the absence of any commercial or financial relationships that could be construed as a potential conflict of interest.

## Abbreviations

CPT1A: carnitine palmitoyltransferase 1A
CWB: community well-being index
HWE: Hardy-Weinberg equilibrium
LRTI: lower respiratory tract infection
OM: otitis media
PTB: preterm birth
RSV: respiratory syncytial virus.
