# Supplementary material for: Association of the CPT1A p.P479L Metabolic Gene Variant With Childhood Respiratory and Other Infectious Illness in Nunavut
Source: Front Pediatr. 2021 Jul 6;9:678553. doi: 10.3389/fped.2021.678553 (PMC8290072; doi:10.3389/fped.2021.678553)
Supplement: Supplementary file 1 [file Table_1.DOCX]

Supplementary Table 1: Pairwise correlation coefficients between outcomes and variables

|  | **LRTI <5yrs** | **RSV <5yrs** | **OM <5yrs** | **GE <5yrs** | **Dental <5yrs** | **p.P479L Hmz** | **p.P479L Het** | **CWB** | **Iqaluit** | **Male** | **PTB** | | **CHDs** | | **Other CAs** | **Mat. Smk** | **BF ≥6mth** | **Food**  **Insecure** |
| --- | --- | --- | --- | --- | --- | --- | --- | --- | --- | --- | --- | --- | --- | --- | --- | --- | --- | --- |
| **LRTI <5yr** | 1.00 |  |  |  |  |  |  |  |  |  |  | |  | |  |  |  |  |
| **RSV <5yr** | 0.492^a^ | 1.00 |  |  |  |  |  |  |  |  |  | |  | |  |  |  |  |
| **OM <5yr** | 0.080^a^ | 0.053^b^ | 1.00 |  |  |  |  |  |  |  |  | |  | |  |  |  |  |
| **GE <5yr** | 0.091^a^ | 0.041^b^ | 0.146^a^ | 1.00 |  |  |  |  |  |  |  | |  | |  |  |  |  |
| **Dental < 5yr** | 0.062^b^ | 0.012 | 0.070^b^ | 0.037 | 1.00 |  |  |  |  |  |  | |  | |  |  |  |  |
| **p.P479L Hmz** | 0.147^a^ | 0.052^b^ | 0.094^b^ | 0.098^a^ | 0.093^a^ | 1.00 |  |  |  |  |  | |  | |  |  |  |  |
| **p.P479LHet** | -0.118^a^ | -0.035 | -0.039 | -0.069^b^ | -0.028 | -0.870^a^ | 1.00 |  |  |  |  | |  | |  |  |  |  |
| **CWB** | -0.053^b^ | -0.012 | -0.221^a^ | -0.122^a^ | -0.185^a^ | -0.296^a^ | 0.181^a^ | 1.00 |  |  |  | |  | |  |  |  |  |
| **Iqaluit** | 0.014 | 0.018 | -0.235^a^ | -0.064^b^ | -0.109^a^ | -0.217^a^ | 0.121^a^ | 0.790^a^ | 1.00 |  |  | |  | |  |  |  |  |
| **Male** | 0.032 | -0.006 | -0.000 | 0.039 | 0.001 | 0.006 | 0.002 | -0.018 | -0.017 | 1.00 |  | |  | |  |  |  |  |
| **PTB** | 0.144^a^ | 0.046^b^ | -0.041^b^ | -0.020 | -0.018 | 0.008 | -0.046^b^ | -0.034 | -0.008 | 0.0479^b^ | 1.00 | |  | |  |  |  |  |
| **CHD** | 0.131^a^ | 0.044^b^ | 0.020 | -0.012 | 0.046^b^ | 0.014 | -0.044^b^ | -0.042^b^ | -0.024 | -0.053^b^ | 0.087^a^ | | 1.00 | |  |  |  |  |
| **Other CAs** | 0.087^a^ | 0.032 | 0.025 | 0.0001 | 0.003 | 0.033 | -0.046^b^ | -0.005 | -0.014 | 0.044^b^ | 0.056^b^ | | 0.032 | | 1.00 |  |  |  |
| **Mat. Smk** | 0.063^b^ | 0.024 | 0.041 | 0.007 | 0.040 | 0.110^a^ | -0.033 | -0.150^a^ | -0.142^a^ | 0.021 | 0.025 | | 0.005 | | 0.006 | 1.00 |  |  |
| **BF ≥6mths** | -0.112^a^ | -0.019 | -0.004 | -0.039 | -0.041^b^ | -0.034 | 0.032 | 0.029 | -0.020 | -0.035 | -0.077^a^ | -0.035 | | -0.018 | | -0.076^b^ | 1.00 |  |
| **Food Insecure** | 0.063^b^ | 0.038 | 0.027 | 0.030 | 0.028 | 0.147^a^ | -0.101^a^ | -0.164^a^ | -0.154^a^ | 0.014 | -0.006 | | 0.026 | | -0.038 | 0.146^a^ | 0.016 | 1.00 |

LRTI: lower respiratory tract infection, RSV: Respiratory syncytial virus, OM: Otitis media, GE: Gastroenteritis, Dental: major dental interventions (extractions, restorations, surgeries). CPT1A: carnitine palmitoyltransferase 1A, p.P479L Hmz (LL): homozygous for CPT1A p.P479L variant, p.P479L Het (PL): heterozygous for the CPT1A p.P479L variant, CWB: community well-being index, PTB: preterm birth (<37weeks gestation), CHD: presence of congenital heart defect, Other CAs: presence of other major congenital anomalies, Mat. Smk: postnatal maternal smoking, BF6mths+: breastfeeding 6 months or longer, FI: Food insecurity.

^a^significant at p=0.05 using Bonferroni correction for multiple testing

^b^significant at p=0.05
